# Translating Research Evidence Into Marketplace Application: Cohort Study of Internet-Based Intervention Platforms for Perinatal Depression

**DOI:** 10.2196/42777

**Published:** 2023-04-17

**Authors:** Zhen Zeng, Jiale Peng, Lu Liu, Wenjie Gong

**Affiliations:** 1 HER Team and XiangYa School of Public Health Central South University Changsha, Hunan China; 2 Institute of Applied Health Research University of Birmingham Birmingham United Kingdom; 3 Department of Psychiatry University of Rochester Rochester, NY United States

**Keywords:** cohort, digital health, internet-based intervention platform, mhealth, perinatal depression, quality assessment

## Abstract

**Background:**

Internet-based intervention platforms may improve access to mental health care for women with perinatal depression (PND). Though the majority of platforms in the market lack an evidence base, a small number of them are supported by research evidence.

**Objective:**

This study aims to assess the current status of internet-based PND intervention platforms supported by published evidence, understand the reasons behind the disappearance of any of these previously accessible platforms, examine adjustments made by those active platforms between research trials and market implementation, and evaluate their current quality.

**Methods:**

A cohort of internet-based PND intervention platforms was first identified by systematic searches in multiple academic databases from database inception until March 26, 2021. We searched on the World Wide Web and the iOS and Android app stores to assess which of these were available in the marketplace between April and May 2021. The basic characteristics of all platforms were collected. For inaccessible platforms, inquiries were made via email to the authors of publications to determine the reasons for their unavailability. We compared the intervention-related information of accessible platforms in the marketplace with that reported in original publications and conducted quality assessments using the App Evaluation Model of the American Psychiatric Association. Fisher exact tests were used to compare the functional characteristics in publications of available and unavailable platforms and to investigate potential associations between functional adjustments or quality indices and platform survival time.

**Results:**

Out of 35 platforms supported by research evidence, only 19 (54%) were still accessible in the marketplace. The main reason for platforms disappearing was the termination of research projects. No statistically significant differences were found in functional characteristics between available and unavailable platforms. A total of 18 (95%) platforms adapted their core functions from what was reported in related publications. The adjustments included changes to intervention methods (11/19, 58%), target population (10/19, 53%), human resources for intervention support (9/19, 47%), mood assessment and monitoring (8/19, 42%), communication modality (4/19, 21%), and platform type (2/19, 11%). Quality issues across platforms included low frequency of update, lack of crisis management mechanism, poor user interactivity, and weak evidence base or absence of citation of supporting evidence. Platforms that survived longer than 10 years had a higher tendency to use external resources from third parties compared to those that survived less than 10 years (*P*=.04). No significant differences were found for functional adjustments or other quality indices.

**Conclusions:**

Internet-based platforms supported by evidence were not effectively translated into real-world practice. It is unclear if adjustments to accessible platforms made during actual operation may undermine the proven validity of the original research. Future research to explore the reasons behind the success of the implementation of evidence-based platforms in the marketplace is warranted.

## Introduction

Perinatal depression (PND) is one of the most common complications of childbirth [1], with a global prevalence of 11.9% (95% CI 11.4-12.5) [2]. If left untreated, PND can have serious consequences for both mothers and their newborns [3]. Despite the availability of evidence-based interventions, women with PND face multiple barriers to accessing these health services through conventional in-person visits, including stigma, inadequate local health care resources, the burden of infant care, and social restrictions during the COVID-19 pandemic [4,5]. Internet-based platforms that were designed to deliver a variety of interventions have the potential to improve accessibility and uptake of mental health care due to their flexibility, protection of privacy, affordability, and accessibility [6-9].

One problem in this burgeoning area is that the vast majority of platforms available in the marketplace are not supported by any scientific evidence of their effectiveness. Larsen and colleagues [10] assessed 73 mental health apps available in 2019 and found that only 2 provided original research evidence. Another recent study evaluated 14 perinatal mental health assessment apps on the market, and only 1 app cited a relevant feasibility study [11]. On the other hand, there is a considerable body of research evidence supporting the feasibility and effectiveness of internet-based PND platforms using a variety of intervention methods, including health education, mood monitoring, peer support, and psychotherapy [6-8,12]. The reasons behind this paradox are unclear. In particular, it would be interesting to examine if platforms supported by literature evidence are effectively implemented in the real world and, if not, what the explanations may be.

In this study, we aimed to (1) identify a cohort of internet-based platforms (websites or apps) for which at least some research evidence was found in the literature; (2) track down their current statuses in the marketplace; and (3) understand the reasons behind the disappearance of previously accessible platforms. We also aimed to examine any adjustments made by these platforms to the functionality reported in the research evidence and assess the quality of currently accessible platforms against the App Evaluation Model of the American Psychiatric Association (APA’s model) [13].

## Methods

### Study Cohort

The study cohort consisted of internet-based PND intervention platforms supported by research evidence. In this study, we defined “platform” as an internet-based technology that offers a variety of mental health interventions or services to users, accessible through mobile apps or websites on smartphones, tablets, or computers. We excluded text messaging interventions that use SMS text messaging or technology-enabled services accessed through smartwatches or virtual reality headsets from our definition. We searched PubMed, Web of Science, Cochrane Library, Embase, CNKI, Wanfang, and VIP databases using “perinatal,” “depression,” “internet,” “intervention,” and their extended terms as keywords, from database inception until March 26, 2021 (search terms are shown in the Multimedia Appendix 1). We included platforms mentioned in the retrieved literature if they met the following inclusion criteria: (1) platform was designed to intervene PND; (2) platform type was websites or apps; (3) the original studies reported the effectiveness or feasibility of the platforms; and (4) the original studies were published in Chinese or English. The platforms mentioned in reviews, conference abstracts, or letters were excluded. For eligible platforms, 2 researchers independently extracted data from the corresponding publications, including the basic information of literature (authors and year of publication) and the types and names of platforms, to form a platform database. We also collected and tabulated the intervention features of the platforms described in the literature, including the target population, methods used, communication modality, and human resources provided for support, and whether the platform has a mood assessment function. However, some of the publications did not specify the platform used in their research or provide sufficient details about the intervention. We sent emails to the authors of the relevant papers with a brief questionnaire that included questions about the platform’s name, availability status, and, if applicable, its app download or website address. If the platform was no longer available, we asked for the reasons and the date of the outage. We gave priority to corresponding authors, followed by first and second authors. If we did not receive a response to our initial email, we sent a reminder email at 5-day intervals, up to a maximum of 3 times per author. Despite attempts to contact the authors for additional information, no response was received. As a result, these platforms were not included in our analysis.

### Follow-up Data Collection

We followed up with each eligible platform to collect relevant information regarding their real-world operations. Using information from the platform database as keywords, multiple searches were performed in the Google search engine, Apple App Store (iOS), and Google Play store (Android) between April and May 2021 to identify the corresponding platforms in the marketplace. The current status (accessible or not) of the platform was recorded. For platforms currently accessible, 2 researchers then independently collected market-related data, including platform type (website or app), estimated operating time (period between the launch of the platform and data extraction in this study), and language. For platforms that were no longer accessible at the time of follow-up, emails were addressed to the authors of the related papers, inquiring about the reason for platform inaccessibility. Our approach to contacting authors is the same as previously outlined.

### Data Analysis

The data collected in the literature and marketplace were analyzed using descriptive statistics, calculating the percentages, averages, and ranges to describe the characteristics of the platforms. We used Fisher exact tests to assess the functional differences between available and unavailable platforms, as well as potential associations between functional adjustments or quality indices and platform survival time. The statistical significance was set at α=.05. All statistical tests were conducted using IBM SPSS Statistics (version 25.0).

For platforms currently accessible, we assessed the functional adjustments by comparing intervention-related information in real-world operations with the original publications, including platform type, intervention methods, target population, human resources to support interventions, communication modality, and mood assessment and monitoring functions. The quality of accessible platforms was evaluated based on the APA’s model, which was developed by the American Psychiatric Association to assist clinicians and individuals in reviewing the risks and benefits of app use. APA’s model comprises the following 5 levels of evaluation: access and background, privacy and security, clinical foundation, usability, and data integration toward the therapeutic goal. Each level contains a series of objective indicators that address the critical standards for app evaluation as identified by mobile health leaders [14]. The APA’s model is distinguished from other app evaluation frameworks by its comprehensiveness, flexibility, and potential for adoption in various contexts. This allows multiple stakeholders to tailor the evaluation process to meet their specific needs. In this study, we selected key indicators from each level of the APA’s model that were applicable to both websites and apps to form a quality assessment checklist (see Multimedia Appendix 2). All accessible platforms were examined thoroughly based on this checklist by browsing the websites’ and apps’ interfaces. The researchers conducting the assessment were fully trained prior to the start of the study to ensure that the relevant information could be accurately extracted and interpreted. Any disagreements were discussed until a consensus was reached.

## Results

### Cohort Profile

**Figure 1:**
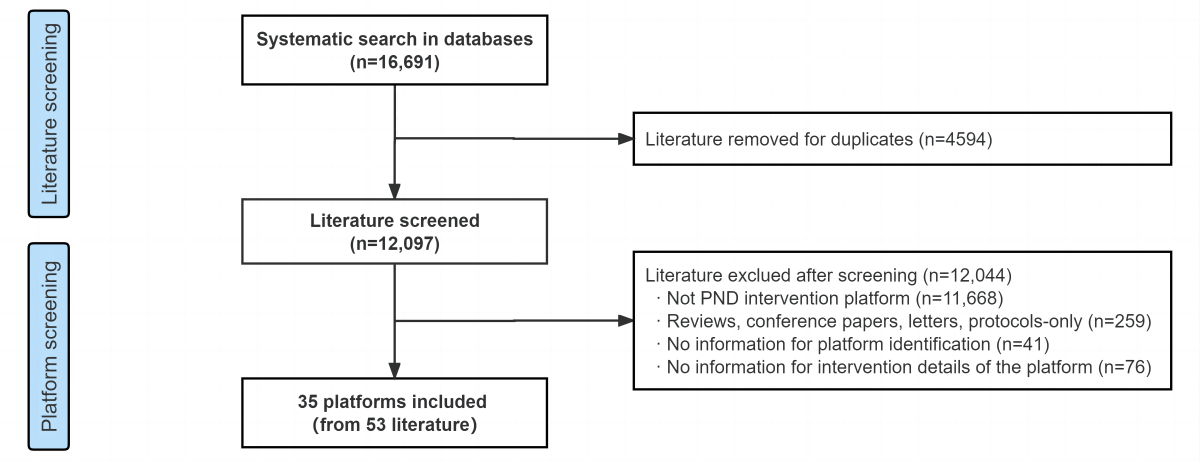
Flowchart of platforms included for assessment. PND: perinatal depression.

After excluding duplicates (n=4594), a total of 12,097 publications were retrieved from academic databases. A cohort of 35 platforms derived from 53 original research papers was established following the literature screening. A detailed platform database can be seen in Multimedia Appendix 3. A flowchart of platforms included for follow-up assessment can be found in Figure 1.

### Current Status of Platforms

Of the 35 platforms supported by published literature, only 19 were currently accessible, with an average estimated operating time of 12 years. The authors of 7 of the 16 inaccessible platforms provided the precise out-of-service time, from which we calculated an average estimated operating time of 2.1 years. The basic characteristics of 35 platforms can be found in Table 1.

**Table 1:** Basic characteristics of perinatal depression internet-based intervention platforms.

		Platforms included (N=35)	Platforms available (n=19)	Platforms unavailable (n=16)
**Platform type, n**				
	Website only	19	11	8
	App only	13	5	8
	Both website and app	3	3	0
**Survival duration (years)**				
	Mean	9.2^a^	12.0	2.1^a^
**Survival duration, n**				
	0-5	12	5	7^a^
	5-10	3	3	0
	10-15	5	5	0
	≥15	6	6	0
**Language** **type, n**				
	English	20	15	5
	Others	12	4	8
	Unknown	3	0	3
**Language, n**				
	Single	23	12	11
	Multiple	9	7	2
	Unknown	3	0	3

^a^Data for unavailable platforms were calculated based on the platforms to which the author replied to the email (n=7).

### Reasons Why Some Platforms Were No Longer Available on the Internet

The authors of 10 of the 16 inaccessible platforms replied to our emails and confirmed the reasons for unavailability were the termination of the research project (n=9) and a technology incompatibility issue (n=1). Table 2 compared the functional characteristics mentioned in relevant publications for all the platforms. Descriptive data show that nonavailable platforms tended to focus more on antenatal populations, offer less synchronized communication, provide less human support for the intervention, and be more app based compared to available platforms, although statistical analysis did not reveal significant differences.

**Table 2:** Functional characteristics of included platforms in literature.

Functional characteristics		Platforms available (n=19), n (%)	Platforms unavailable (n=16), n (%)	*P* value
**Intervention method**				>.99
	Psychotherapy (CBT^a^, BA^b^, PST^c^, etc)	9 (47.4)	7 (43.7)	
	Nonpsychotherapy	10 (52.6)	9 (56.3)	
**Target population**				.25
	Antenatal	5 (26.3)	9 (56.3)	
	Postnatal	9 (47.4)	5 (31.2)	
	Perinatal	5 (26.3)	2 (12.5)	
**Human support for interventions**				.32
	Yes	11 (57.9)	6 (37.5)	
	No	8 (42.1)	10 (62.5)	
**Mood monitoring and assessment**				.50
	Yes	6 (31.6)	7 (43.7)	
	No	13 (68.4)	9 (56.3)	
**Synchronous communication**				.24
	Yes	6 (31.6)	2 (12.5)	
	No	13 (68.4)	14 (87.5)	
**Platform type**				.11
	Website only	13 (68.4)	8 (50)	
	App only	4 (21.1)	8 (50)	
	Both website and app	2 (10.5)	0 (0)	

^a^CBT: cognitive behavioral therapy.

^b^BA: behavioral activation therapy.

^c^PST: problem solving therapy.

### Functional Adjustments Between Literature Evidence and Market Operation

**Figure 2:**
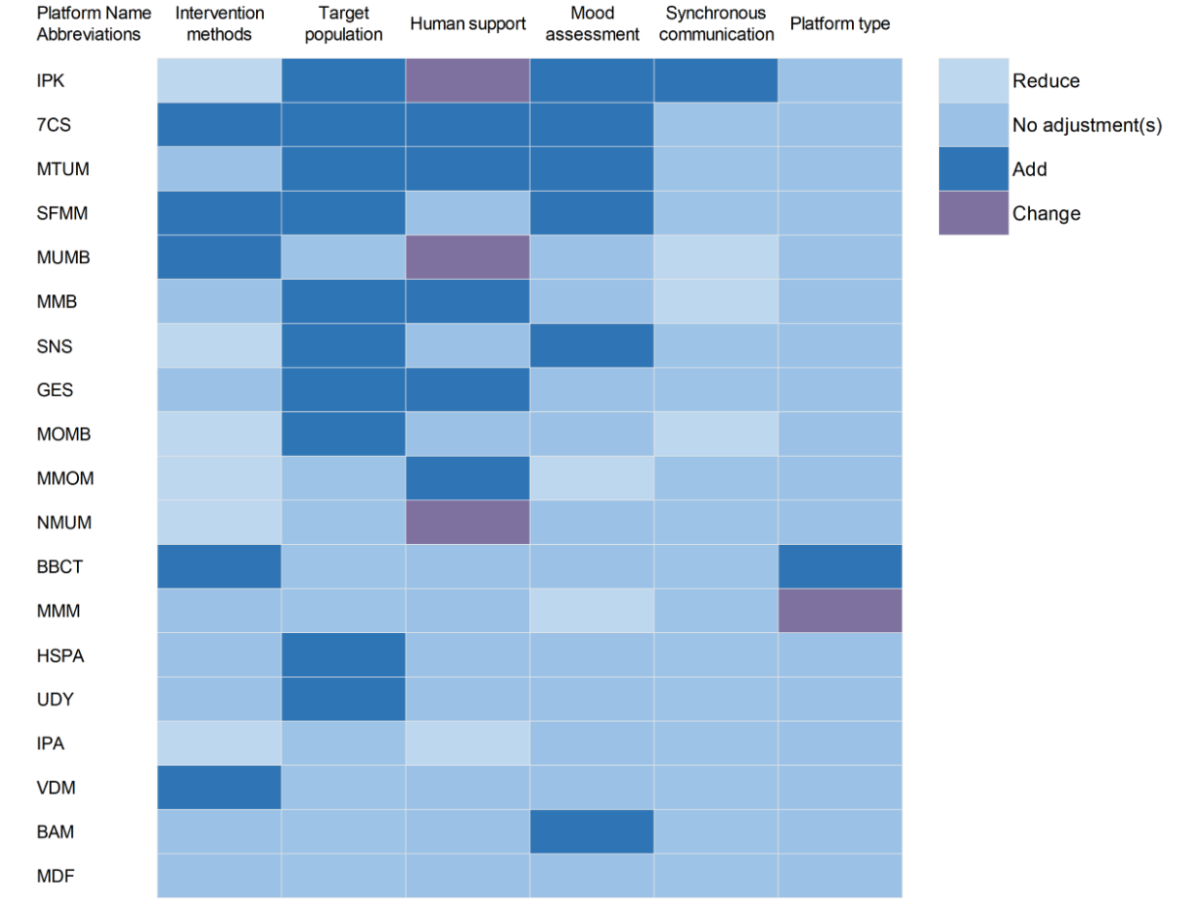
Functional adjustments of available platforms in the market compared to those reported in the original publications. 7CS: 7 Cups; BAM: Be a mom; BBCT: Babycenter; GES: Ginger Emotional Support; HSPA: Headspace; IPA: iParent; IPK: Internetpsykiatri; MDF: Mindful-ouderschap; MMB: Mindful Mood Balance; MMM: Mamma Mia; MMOM: Mindmom; MOMB: MomMoodBooste; MTUM: MUMentum; MUMB: MumMoodBooster; NMUM: Netmums; SFMM: Strongest FamiliesManaging Our Mood (MOM); SNS: Sunnyside; UDY: Udaya; VDM: Veedamom.

A total of 18 of the 19 still-accessible platforms adjusted their actual functions in the market in comparison to those reported in the publications (Figure 2). Eight platforms maintained the original designs for intervention methods, while 5 platforms introduced new methods of mood diary (n=2), mindfulness meditation (n=2), and cognitive behavioral therapy (CBT; n=1). Six platforms removed their original intervention features of CBT (n=3), behavioral activation therapy (n=2), and mood diary (n=1). Ten platforms have a wider target population than what was originally studied. Nine platforms had human support for intervention that differed from the original literature. Among them, 3 platforms with no human support planned in the original studies added clinicians, psychotherapists, and other professionals in the real-world setting; 2 platforms provided additional types of human support; 1 platform was intended to provide human support but did not do so in practice; and 3 platforms changed the type of human support. Eight platforms adapted the function of mood assessment and monitoring from the original literature, with 6 platforms adding mood assessment and 2 platforms removing the mood assessment module in actual operation. The majority of platforms retained their original communication modality, with only 4 making adjustments. Three of these platforms removed the planned synchronous communication, and 1 platform added synchronous mode. Seventeen platforms ran in the same type as designed in the original literature, with only 1 platform adding a new operational type and 1 switching from website to app. No statistically significant differences were found upon further exploration of the association between functional adjustments and platform survival time. Additional details can be found in Multimedia Appendix 4.

### Quality Assessment Based on APA’s Model

Detailed results of the quality assessment of all accessible platforms following the APA’s model can be seen in Multimedia Appendix 5.

#### Access and Background

All platforms had specific developers, including private companies (n=5), academic organizations (n=5), and medical health providers (n=3). In addition, there were also 6 platforms with multiple types of developers, 5 of which were led by academic organizations in collaboration with government, private companies, and medical health providers respectively, and the remaining 1 was led by a private company in cooperation with individuals that had living experience. Eleven of the 19 platforms were exclusively accessible through websites; 5 were only delivered with apps; and 3 offered both. Two of the 8 platforms offering apps were exclusive to either Android or iOS devices, while 6 were available on both devices. Only 6 platforms have been updated within 6 months. Twelve of the 19 platforms were entirely free, while the remaining 7 charged, mostly on a weekly, monthly, or quarterly basis, according to the services, with an average weekly cost of US $14.17. Two platforms would charge extra for personalized services on top of the base rate.

#### Privacy and Security

The majority of platforms disclosed user-related specifications; 14 platforms included a privacy policy, and 13 provided terms of use. A total of 16 platforms claimed that they collect, use, or transmit users’ data. In terms of resource use, 13 platforms used third-party resources. Of the 19 platforms, 8 had crisis management mechanisms, of which 5 were passive, that is, they offered information such as helplines to be used when necessary. Three of them adopted active management procedures, that is, the platforms would proactively send out alerts and offer assistance to users in need.

#### Clinical Foundation and Usability

Only 8 platforms provided research-based references. All platforms had explicit intervention methods, with health education (n=17) being the most commonly used, followed by mindfulness meditation (n=6), CBT (n=5), social support (n=5), and mood diary (n=3), respectively. A total of 13 platforms provided users with human support during the intervention, 6 of which were given by professionals (doctors, psychotherapists, etc), 5 by nonprofessionals, and 2 by a combination of both sorts. Ten platforms offered users feedback. There were 10 platforms that assessed users’ mood, but only 2 of them specified the method of assessment (Edinburgh Postnatal Depression Scale and Patient Health Questionnaire-9). Regarding platform engagement styles, 4 platforms used only image-text presentation mode; 14 platforms integrated audio, video, animation, and other forms; and only 1 platform employed artificial intelligence technology, albeit ineffectively.

#### Data Integration Toward Therapeutic Goal

Ten platforms offered data export options to users. Six platforms provided users in need with referral information, 4 of which offered offline visits and 2 web-based visits. However, only 2 platforms were capable of directly integrating user data into existing health care systems.

We investigated the association between quality indices and platform survival time and found that platforms lasting over 10 years had a significantly higher tendency to use external resources from third parties than those lasting less than 10 years (*P*=.04). However, we did not find any statistically significant differences in platform survival time for other quality indices. More information can be found in Multimedia Appendix 6.

## Discussion

### Principal Findings

With a cohort of websites and apps that have been reported in peer-reviewed literature, we examined for the first time how internet-based PND intervention platforms supported by published evidence operated in the marketplace. We found that nearly half of these platforms were no longer accessible, with an average estimated operating time of 2.1 years. The results are consistent with the current state of the mobile health market, where every 2.9 days a clinical app related to depression becomes unavailable in the App store [15]. However, unlike platforms on the market where user engagement was one of the most important factors for their sustainability [16,17], the main reason why the platforms identified in our study were no longer available was the termination of the research project. The fact that these platforms in literature might have never operated outside of the research setting is a useful reminder of the challenges in translating evidence-based platforms into market implementation. Most of the still-available platforms (14/19) have been in operation for more than 5 years, indicating that platforms that entered the market early and survived may have attracted a large number of users and are more capable of retaining user engagement, whereas new platforms will face a “red ocean” market with nearly saturated demand and will have a more difficult time surviving. We further compared the differences in functional characteristics between unavailable and available platforms in the original literature and found that unavailable platforms were less likely to use synchronous communication and less likely to provide manual assistance for interventions. These features are often associated with a better user experience. This suggests that the design of unavailable platforms may not have been well-suited to the actual needs of the market, which may have contributed to their difficulty in surviving.

Compared with the original publications, many platforms adjusted the core elements of the interventions in actual operations. Changes in intervention methods were the most common adjustments, and the main adding features were those that increased user engagement, such as mood diary [12,18-20], mindfulness, and meditation [21,22]. These features are popular methods in internet-based platforms, because not only do they help to improve users’ outcomes and experience; they are also often implemented in a user- and technology-driven way without additional human operating costs [23]. Five platforms removed the originally planned psychotherapy, such as CBT and behavioral activation therapy, probably because the implementation of these methods requires long-term, high-intensity investment from mental health specialists, which is difficult to maintain in actual operation [24]. Those platforms that removed synchronous communication from their original design may have done so based on similar considerations. We also found that half of the platforms have a larger range of target population in actual operation than what was studied in the research scenario. This may be because a wider user base is more conducive to improving the platform’s market competitiveness. Six platforms added mood assessment functions, possibly for mood monitoring and increasing user engagement [25]. Nine platforms have adjusted their staffing provision for interventions, with 5 platforms adding human support and 3 have changed the type of supporting personnel. This indicated that although self-management is a major feature and selling point of internet-based intervention platforms, providing a certain level of staff support may still be the mainstream of market demand [12]. It is clear that research-validated PND intervention platforms have continued to evolve in response to market demand and resource limitations. However, these adjustments may, in turn, have compromised the proven effectiveness or feasibility of the platforms.

A thorough quality assessment of the 19 platforms currently accessible revealed some common problems across each dimension of the APA’s model. Only about one-third of platforms had been updated in the past 6 months, which is consistent with Spadaro et al’s [11] report that focused on platforms available in the market. Internet-based intervention platforms that fail to maintain a high frequency of content updates or upgrades will struggle to stay competitive in a crowded market. For apps in particular, in addition to content refreshing, regular software updates must be ensured to maintain usability after updates to iOS and Android system [26]. Another prevalent problem among these platforms is the lack of crisis management. Of the 19 platforms assessed in this study, 8 had crisis management mechanisms, and only 3 of them will actively present instance warning messages and provide help-seeking information to users upon monitoring signs of suicidal ideation or self-harm. This will pose significant risks to both users and the platforms, as some women with PND may have a higher risk of thoughts or behaviors of self-harm and suicide [27]. Therefore, more efforts should be put into platform design to establish monitoring mechanisms and provide timely and appropriate care. Although most (16/19, 84%) platforms claimed to collect users’ data, only 10 platforms provided feedback to users (including but not limited to mood assessment results). It indicated that nearly half of the platforms still struggled with inadequate user interaction, which may hamper users’ engagement. The proportion of platforms that offered data export options to users in this study (10/19, 53%) is higher than Spadaro et al’s [11] finding (3/14, 21%), which may be because platforms based on published evidence might have a higher level of involvement of medical practitioners in the design and operation, thus focusing more on the integration of care. However, among the 10 platforms with data export services, only 2 integrated users’ data into the health care system, suggesting that, though the importance of integrated care is recognized by researchers, its translation into practice is patchy. Previous studies showed that platforms available in the marketplace often lack quality evidence [10,11]. In this study, even though scientific evidence was readily available, less than half (8/19, 42%) of platforms actively mentioned relevant research evidence to support their operations.

### Limitations

This study has some limitations. Although this paper did not include a systematic review, we employed a systematic search process to identify eligible platforms. We were unable to obtain detailed information on platforms between literature publication and our evaluation (eg, how many times the platform has adjusted its functions and when the adjustments occurred). But in contrast to previous studies that only took snapshots of market available apps, this study provided a longitudinal perspective by focusing on a cohort of internet-based platforms supported by peer-reviewed literature and tracking their real-world status. This study did not examine subjective indicators of quality, such as ease of use. However, we have conducted a comprehensive, objective quality assessment of all platforms based on the APA’s model to obtain a complete understanding of the quality of internet-based platforms.

### Conclusions

Internet-based intervention platforms for PND are in a period of rapid development, but this study shows that many evidence-based platforms have poor sustainability in the marketplace. Despite a variety of functionality adjustments in actual operation, most platforms still had quality concerns that were not discussed in the research design phase. The ultimate goal of intervention platform development is to be implemented in the real world. It is clear that feasibility or effectiveness studies within the research context alone are insufficient for this goal. Further research from the perspective of implementation science would be required to evaluate the long-term effectiveness of the platform in a real-world setting, identify the barriers and facilitators of the implementation, and inform platform design in the future.

## Appendix

Multimedia Appendix 1 Search terms in each database.

Multimedia Appendix 2 Quality assessment checklist.

Multimedia Appendix 3 Platform database.

Multimedia Appendix 4 The associations between functional adjustments and survival time of internet-based perinatal depression intervention platforms (n=19).

Multimedia Appendix 5 Quality assessment of internet-based perinatal depression intervention platforms against the American Psychiatric Association’s model (n=19).

Multimedia Appendix 6 The associations between quality indices and survival time of internet-based perinatal depression intervention platforms (n=19).

## Abbreviations

APA’s model: App Evaluation Model of the American Psychiatric Association
CBT: cognitive behavioral therapy
PND: perinatal depression

## Data Availability

The data sets generated and analyzed during this study are available from the corresponding author on reasonable request.

## References

[1] Gavin Ni, Gaynes Bn, Lohr Kn, Meltzer-Brody S, Gartlehner G, Swinson T (2005). Perinatal depression: a systematic review of prevalence and incidence. Obstet Gynecol.

[2] Woody CA, Ferrari AJ, Siskind DJ, Whiteford HA, Harris MG (2017). A systematic review and meta-regression of the prevalence and incidence of perinatal depression. J Affect Disord.

[3] Dagher RK, Bruckheim HE, Colpe LJ, Edwards E, White DB (2021). Perinatal Depression: Challenges and Opportunities. J Womens Health (Larchmt).

[4] Lukas H, Xu C, Yu Y, Gao W (2020). Emerging telemedicine tools for remote COVID-19 diagnosis, monitoring, and management. ACS Nano.

[5] Iturralde E, Hsiao CA, Nkemere L, Kubo A, Sterling SA, Flanagan T, Avalos LA (2021). Engagement in perinatal depression treatment: a qualitative study of barriers across and within racial/ethnic groups. BMC Pregnancy Childbirth.

[6] Ashford MT, Olander EK, Ayers S (2016). Computer- or web-based interventions for perinatal mental health: a systematic review. J Affect Disord.

[7] Lee EW, Denison FC, Hor K, Reynolds RM (2016). Web-based interventions for prevention and treatment of perinatal mood disorders: a systematic review. BMC Pregnancy Childbirth.

[8] Hussain-Shamsy N, Shah A, Vigod SN, Zaheer J, Seto E (2020). Mobile health for perinatal depression and anxiety: scoping review. J Med Internet Res.

[9] van den Heuvel JF, Groenhof TK, Veerbeek JH, van Solinge WW, Lely AT, Franx A, Bekker MN (2018). eHealth as the next-generation perinatal care: an overview of the literature. J Med Internet Res.

[10] Larsen ME, Huckvale K, Nicholas J, Torous J, Birrell L, Li E, Reda B (2019). Using science to sell apps: evaluation of mental health app store quality claims. NPJ Digit Med.

[11] Spadaro B, Martin-Key NA, Funnell E, Bahn S (2022). mHealth solutions for perinatal mental health: scoping review and appraisal following the mHealth index and navigation database framework. JMIR Mhealth Uhealth.

[12] Milgrom J, Danaher BG, Gemmill AW, Holt C, Holt CJ, Seeley JR, Tyler MS, Ross J, Ericksen J (2016). Internet cognitive behavioral therapy for women with postnatal depression: a randomized controlled trial of MumMoodBooster. J Med Internet Res.

[13] The App Evaluation Model. American Psychiatric Association.

[14] Lagan S, Emerson MR, King D, Matwin S, Chan SR, Proctor S, Tartaglia J, Fortuna KL, Aquino P, Walker R, Dirst M, Benson N, Myrick KJ, Tatro N, Gratzer D, Torous J (2021). Mental health app evaluation: updating the American Psychiatric Association's framework through a stakeholder-engaged workshop. Psychiatr Serv.

[15] Larsen ME, Nicholas J, Christensen H (2016). Quantifying App Store dynamics: longitudinal tracking of mental health apps. JMIR Mhealth Uhealth.

[16] Baumel A, Muench F, Edan S, Kane JM (2019). Objective user engagement with mental health apps: systematic search and panel-based usage analysis. J Med Internet Res.

[17] Borghouts J, Eikey E, Mark G, De Leon C, Schueller SM, Schneider M, Stadnick N, Zheng K, Mukamel D, Sorkin DH (2021). Barriers to and facilitators of user engagement with digital mental health interventions: systematic review. J Med Internet Res.

[18] Gold KJ, Boggs ME, Kavanaugh KL (2021). MOMSonLINE: lessons learned from a feasibility RCT of online support for mothers bereaved by perinatal loss. Omega (Westport).

[19] BabyCenter.

[20] MumMoodBooster: an online treatment for antenatal depression compared to best-practice alternatives.

[21] Prasad V (2018). Efficacy of an electronic application to moderate symptoms of postpartum depression and improve postpartum well-being: a pilot study. Texas A&M University-Corpus Christi.

[22] Veedamom. App Store.

[23] Spijkerman MP, Pots WT, Bohlmeijer ET (2016). Effectiveness of online mindfulness-based interventions in improving mental health: a review and meta-analysis of randomised controlled trials. Clin Psychol Rev.

[24] Shafran R, Clark DM, Fairburn CG, Arntz A, Barlow DH, Ehlers A, Freeston M, Garety PA, Hollon SD, Ost LG, Salkovskis PM, Williams JMG, Wilson GT (2009). Mind the gap: improving the dissemination of CBT. Behav Res Ther.

[25] Li Y, Guo Y, Hong YA, Zeng Y, Monroe-Wise A, Zeng C, Zhu M, Zhang H, Qiao J, Xu Z, Cai W, Li L, Liu C (2022). Dose-response effects of patient engagement on health outcomes in an mHealth intervention: secondary analysis of a randomized controlled trial. JMIR Mhealth Uhealth.

[26] Wisniewski H, Liu G, Henson P, Vaidyam A, Hajratalli NK, Onnela J, Torous J (2019). Understanding the quality, effectiveness and attributes of top-rated smartphone health apps. Evid Based Ment Health.

[27] Mangla K, Hoffman MC, Trumpff C, O'Grady S, Monk C (2019). Maternal self-harm deaths: an unrecognized and preventable outcome. Am J Obstet Gynecol.

