# Supplementary material for: Translating Research Evidence Into Marketplace Application: Cohort Study of Internet-Based Intervention Platforms for Perinatal Depression
Source: J Med Internet Res. 2023 Apr 17;25:e42777. doi: 10.2196/42777 (PMC10152328; doi:10.2196/42777)
Supplement: Multimedia Appendix 1 [file jmir_v25i1e42777_app1.docx]

**Multimedia Appendix 1.** *Search terms in Each Database***.**

The search terms used for literature on internet-based intervention platforms for perinatal depression in each database are as follows:

**PubMed:**

((("Postpartum Period"[Mesh]) OR ("Peripartum Period"[Mesh]) OR (pregnancy[Mesh]) OR (Pregnan*[Title/Abstract]) OR (gestation*[Title/Abstract]) OR (gravid*[Title/Abstract]) OR (antepartum[Title/Abstract]) OR (ante-partum[Title/Abstract]) OR (antenatal[Title/Abstract]) OR (ante-natal[Title/Abstract]) OR (prenatal[Title/Abstract]) OR (pre-natal[Title/Abstract]) OR (peripartum[Title/Abstract]) OR (peri-partum[Title/Abstract]) OR (perinatal[Title/Abstract]) OR (peri-natal[Title/Abstract]) OR (postpartum[Title/Abstract]) OR (post-partum[Title/Abstract]) OR (postnatal[Title/Abstract]) OR (post-natal[Title/Abstract]) OR (puerper*[Title/Abstract]) OR (matern*[Title/Abstract])) AND ((Depression[Mesh]) OR ("Mental Disorders"[Mesh]) OR ("affective symptoms"[Mesh]) OR (depress*[Title/Abstract]) OR (dysthymi*[Title/Abstract]) OR (affective[Title/Abstract]) OR (adjustment[Title/Abstract]) OR (mood[Title/Abstract]))) AND ((((Internet[Mesh]) OR (Computers[Mesh]) OR (Software[Mesh]) OR (Internet*[Title/Abstract]) OR (app*[Title/Abstract]) OR (Computer*[Title/Abstract]) OR (Smartphone*[Title/Abstract]) OR (cellphone*[Title/Abstract]) OR (phone*[Title/Abstract]) OR (Web*[Title/Abstract]) OR (Online*[Title/Abstract]) OR (on-line[Title/Abstract]) OR (Technolog*[Title/Abstract]) OR (Software*[Title/Abstract]) OR (device*[Title/Abstract]) OR (remote*[Title/Abstract])) AND ((Intervention*[Title/Abstract]) OR (therap*[Title/Abstract]) OR (treatment*[Title/Abstract]) OR (assist*[Title/Abstract]) OR (prevent*[Title/Abstract]) OR (prophyla*[Title/Abstract]) OR (education*[Title/Abstract]) OR (manage*[Title/Abstract]) OR (support*[Title/Abstract]) OR (self*[Title/Abstract]) OR (care*[Title/Abstract]) OR (service*[Title/Abstract]) OR (counsel*[Title/Abstract]) OR (consult*[Title/Abstract]) OR (psychotherap*[Title/Abstract]) OR (IPT[Title/Abstract]) OR (CBT[Title/Abstract]) OR (BAT[Title/Abstract]) OR (PST[Title/Abstract]) OR (SFT[Title/Abstract]))) OR (("Internet-based intervention"[Mesh]) OR (Telecommunications[Mesh]) OR (ePortal[Title/Abstract]) OR (e-Portal[Title/Abstract]) OR (etherap*[Title/Abstract]) OR (e-therap*[Title/Abstract]) OR (teletherap*[Title/Abstract]) OR (tele-therap*[Title/Abstract]) OR (telepsych*[Title/Abstract]) OR (tele-psych*[Title/Abstract]) OR ("Mobile Health"[Title/Abstract]) OR ("Health, Mobile"[Title/Abstract]) OR (mHealth[Title/Abstract]) OR (m-health[Title/Abstract]) OR (Telehealth[Title/Abstract]) OR (tele-health[Title/Abstract]) OR (eHealth[Title/Abstract]) OR (e-health[Title/Abstract]) OR ("electronic health"[Title/Abstract]) OR (telemedicine[Title/Abstract]) OR (tele-medicine[Title/Abstract]) OR (mmedicine[Title/Abstract]) OR (m-medicine[Title/Abstract]) OR (telecommunication*[Title/Abstract]) (tele-communication*[Title/Abstract]) OR (ecounsel*[Title/Abstract]) OR (e-counsel*[Title/Abstract]) OR (teleconsult*[Title/Abstract]) OR (tele-consult*[Title/Abstract]) OR (cCBT[Title/Abstract]) OR (iCBT[Title/Abstract]) OR (TECC[Title/Abstract])))

**Embase:**

('puerperium'/exp OR puerperium OR 'perinatal period'/exp OR 'perinatal period' OR 'pregnancy'/exp OR pregnancy OR pregnan?:ti,ab,kw OR gestation?:ti,ab,kw OR gravid?:ti,ab,kw OR antepartum:ti,ab,kw OR 'ante partum':ti,ab,kw OR antenatal:ti,ab,kw OR 'ante natal':ti,ab,kw OR prenatal:ti,ab,kw OR 'pre natal':ti,ab,kw OR peripartum:ti,ab,kw OR 'peri partum':ti,ab,kw OR perinatal:ti,ab,kw OR 'peri natal':ti,ab,kw OR postpartum:ti,ab,kw OR 'post partum':ti,ab,kw OR postnatal:ti,ab,kw OR 'post natal':ti,ab,kw OR puerper?:ti,ab,kw OR matern?:ti,ab,kw) AND ('depression'/exp OR depression OR 'mental disease'/exp OR 'mental disease' OR depress?:ti,ab,kw OR dysthymi?:ti,ab,kw OR affective:ti,ab,kw OR adjustment:ti,ab,kw OR mood:ti,ab,kw) AND ((('internet'/exp OR internet OR 'computer'/exp OR computer OR 'software'/exp OR software OR internet?:ti,ab,kw OR app?:ti,ab,kw OR computer?:ti,ab,kw OR smartphone?:ti,ab,kw OR cellphone?:ti,ab,kw OR 'phone?':ti,ab,kw OR web?:ti,ab,kw OR online?:ti,ab,kw OR 'on line':ti,ab,kw OR technolog?:ti,ab,kw OR software?:ti,ab,kw OR device?:ti,ab,kw OR remote?:ti,ab,kw) AND (intervention?:ti,ab,kw OR therap?:ti,ab,kw OR treatment?:ti,ab,kw OR assist?:ti,ab,kw OR prevent?:ti,ab,kw OR prophyla?:ti,ab,kw OR education?:ti,ab,kw OR manage?:ti,ab,kw OR support?:ti,ab,kw OR self?:ti,ab,kw OR care?:ti,ab,kw OR service?:ti,ab,kw OR counsel?:ti,ab,kw OR consult?:ti,ab,kw OR psychotherap?:ti,ab,kw OR ipt:ti,ab,kw OR cbt:ti,ab,kw OR bat:ti,ab,kw OR pst:ti,ab,kw OR sft:ti,ab,kw)) OR ('web-based intervention'/exp OR 'web-based intervention' OR 'telecommunication'/exp OR telecommunication OR eportal:ti,ab,kw OR 'e portal':ti,ab,kw OR etherap?:ti,ab,kw OR 'e therap?':ti,ab,kw OR teletherap?:ti,ab,kw OR 'tele therap?':ti,ab,kw OR telepsych?:ti,ab,kw OR 'tele psych?':ti,ab,kw OR 'mobile health':ti,ab,kw OR 'health, mobile':ti,ab,kw OR mhealth:ti,ab,kw OR 'm health':ti,ab,kw OR telehealth:ti,ab,kw OR 'tele health':ti,ab,kw OR ehealth:ti,ab,kw OR 'e health':ti,ab,kw OR 'electronic health':ti,ab,kw OR telemedicine:ti,ab,kw OR 'tele medicine':ti,ab,kw OR mmedicine:ti,ab,kw OR 'm medicine':ti,ab,kw OR telecommunication?:ti,ab,kw) AND 'tele communication?':ti,ab,kw) OR ecounsel?:ti,ab,kw OR 'e counsel?':ti,ab,kw OR teleconsult?:ti,ab,kw OR 'tele consult?':ti,ab,kw OR ccbt:ti,ab,kw OR icbt:ti,ab,kw OR tecc:ti,ab,kw))

**Cochrane:**

((("Postpartum Period"[Mesh]) OR ("Peripartum Period"[Mesh]) OR (pregnancy[Mesh]) OR (Pregnan*):ti,ab,kw OR (gestation*):ti,ab,kw OR (gravid*):ti,ab,kw OR (antepartum):ti,ab,kw OR (ante-partum):ti,ab,kw OR (antenatal):ti,ab,kw OR (ante-natal):ti,ab,kw OR (prenatal):ti,ab,kw OR (pre-natal):ti,ab,kw OR (peripartum):ti,ab,kw OR (peri-partum):ti,ab,kw OR (perinatal):ti,ab,kw OR (peri-natal):ti,ab,kw OR (postpartum):ti,ab,kw OR (post-partum):ti,ab,kw OR (postnatal):ti,ab,kw OR (post-natal):ti,ab,kw OR (puerper*):ti,ab,kw OR (matern*):ti,ab,kw) AND ((Depression[Mesh]) OR ("Mental Disorders"[Mesh]) OR ("affective symptoms"[Mesh]) OR (depress*):ti,ab,kw OR (dysthymi*):ti,ab,kw OR (affective):ti,ab,kw OR (adjustment):ti,ab,kw OR (mood):ti,ab,kw)) AND ((((Internet[Mesh]) OR (Computers[Mesh]) OR (Software[Mesh]) OR (Internet*):ti,ab,kw OR (app*):ti,ab,kw OR (Computer*):ti,ab,kw OR (Smartphone*):ti,ab,kw OR (cellphone*):ti,ab,kw OR (phone*):ti,ab,kw OR (Web*):ti,ab,kw OR (Online*):ti,ab,kw OR (on-line):ti,ab,kw OR (Technolog*):ti,ab,kw OR (Software*):ti,ab,kw OR (device*):ti,ab,kw OR (remote*):ti,ab,kw) AND ((Intervention*):ti,ab,kw OR (therap*):ti,ab,kw OR (treatment*):ti,ab,kw OR (assist*):ti,ab,kw OR (prevent*):ti,ab,kw OR (prophyla*):ti,ab,kw OR (education*):ti,ab,kw OR (manage*):ti,ab,kw OR (support*):ti,ab,kw OR (self*):ti,ab,kw OR (care*):ti,ab,kw OR (service*):ti,ab,kw OR (counsel*):ti,ab,kw OR (consult*):ti,ab,kw OR (psychotherap*):ti,ab,kw OR (IPT):ti,ab,kw OR (CBT):ti,ab,kw OR (BAT):ti,ab,kw OR (PST):ti,ab,kw OR (SFT):ti,ab,kw)) OR (("Internet-based intervention"[Mesh]) OR (Telecommunications[Mesh]) OR (ePortal):ti,ab,kw OR (e-Portal):ti,ab,kw OR (iCBT):ti,ab,kw OR (etherap*):ti,ab,kw OR (e-therap*):ti,ab,kw OR (teletherap*):ti,ab,kw OR (tele-therap*):ti,ab,kw OR (telepsych*):ti,ab,kw OR (tele-psych*):ti,ab,kw OR ("Mobile Health"):ti,ab,kw OR ("Health, Mobile" ):ti,ab,kw OR (mHealth):ti,ab,kw OR (m-health):ti,ab,kw OR (Telehealth):ti,ab,kw OR (tele-health):ti,ab,kw OR (eHealth):ti,ab,kw OR (e-health):ti,ab,kw OR ("electronic health"):ti,ab,kw OR (telemedicine):ti,ab,kw OR (tele-medicine):ti,ab,kw OR (mmedicine):ti,ab,kw OR (m-medicine):ti,ab,kw OR (telecommunication*):ti,ab,kw (tele-communication*):ti,ab,kw OR (ecounsel*):ti,ab,kw OR (e-counsel*):ti,ab,kw OR (teleconsult*):ti,ab,kw OR (tele-consult*):ti,ab,kw OR (cCBT):ti,ab,kw OR (TECC):ti,ab,kw))

**Web of science:**

(TS=(Pregnan*) OR TS=(gestation*) OR TS=(gravid*) OR TS=(antepartum) OR TS=(“ante-partum”) OR TS=(antenatal) OR TS=(“ante-natal”) OR TS=(prenatal) OR TS=(“pre-natal”) OR TS=(peripartum) OR TS=(“peri-partum”) OR TS=(perinatal) OR TS=(“peri-natal”) OR TS=(postpartum) OR TS=(“post-partum”) OR TS=(postnatal) OR TS=(“post-natal”) OR TS=(puerper*) OR TS=(matern*)) AND (TS=(“Mental disorders”) OR TS=(depress*) OR TS=(dysthymi*) OR TS=(affective) OR TS=(adjustment) OR TS=(mood)) AND (((TS=(Internet*) OR TS=(app) OR TS=(apps) OR TS=(application*) OR TS=(Computer*) OR TS=(Smartphone*) OR TS=(cellphone*) OR TS=(phone*) OR TS=(Web*) OR TS=(Online*) OR TS=(“on-line”) OR TS=(Technolog*) OR TS=(Software*) OR TS=(device*) OR TS=(remote*)) AND (TS=(Intervention*) OR TS=(therap*) OR TS=(treatment*) OR TS=(assist*) OR TS=(prevent*) OR TS=(prophyla*) OR TS=(education*) OR TS=(manage*) OR TS=(support*) OR TS=(self*) OR TS=(care*) OR TS=(service*) OR TS=(counsel*) OR TS=(consult*) OR TS=(psychotherap*) OR TS=(IPT) OR TS=(CBT) OR TS=(BAT) OR TS=(PST) OR TS=(SFT))) OR (TS=(ePortal) OR TS=(“e-Portal”) OR TS=(etherap*) OR TS=(e-therap*) OR TS=(teletherap*) OR TS=(tele-therap*) OR TS=(telepsych*) OR TS=(tele-psych*) OR TS=(“Mobile Health”) OR TS=(mHealth) OR TS=(“m-health”) OR TS=(Telehealth) OR TS=(“tele-health”) OR TS=(eHealth) OR TS=(“e-health”) OR TS=(“electronic health”) OR TS=(telemedicine) OR TS=(“tele-medicine”) OR TS=(mmedicine) OR TS=(“m-medicine”) OR TS=(telecommunication*) OR TS=(tele-communication*) OR TS=(ecounsel*) OR TS=(e-counsel*) OR TS=(teleconsult*) OR TS=(tele-consult*) OR TS=(cCBT) OR TS=(iCBT) OR TS=(TECC)))

**CNKI:**

(TKA=(产前) or TKA=(分娩) or TKA=(出生) or TKA=(怀孕) or TKA=(孕期) or TKA=(孕妇) or TKA=(孕产妇) or TKA=(妊娠) or TKA=(产妇) or TKA=(围产期) or TKA=(围生期) or TKA=(围生产期) or TKA=(产后) or TKA=(产褥期)) AND (TKA=(抑郁) or TKA=(抑郁症) or TKA=(忧郁) or TKA=(心理) or TKA=(情绪) or TKA=(心境)) AND (TKA=(网络) or TKA=(互联网) or TKA=(网页) or TKA=(网站) or TKA=(线上) or TKA=(在线) or TKA=(电子) or TKA=(移动) or TKA=(app) or TKA=(软件) or TKA=(程序) or TKA=(电脑) or TKA=(计算机) or TKA=(手机) or TKA=(远程) or TKA=(平台)) AND (TKA=(干预) or TKA=(预防) or TKA=(治疗) or TKA=(疗法) or TKA=(医疗) or TKA=(管理) or TKA=(护理) or TKA=(支持) or TKA=(教育) or TKA=(咨询) or TKA=(缓解) or TKA=(监管) or TKA=(问诊) or TKA=(健康))

**Wanfang:**

(主题:(“产前”) or 主题:(“分娩”) or 主题:(“出生”) or 主题:(“怀孕”) or 主题:(“孕期”) or 主题:(“孕妇”) or 主题:(“孕产妇”) or 主题:(“妊娠”) or 主题:(“产妇”) or 主题:(“围产期”) or 主题:(“围生期”) or 主题:(“围生产期”) or 主题:(“产后”) or 主题:(“产褥期”)) AND (主题:(“抑郁”) or 主题:(“抑郁症”) or 主题:(“忧郁”) or 主题:(“心理”) or 主题:(“情绪”) or 主题:(“心境”)) AND (主题:(“网络”) or 主题:(“互联网”) or 主题:(“网页”) or 主题:(“网站”) or 主题:(“线上”) or 主题:(“在线”) or 主题:(“电子”) or 主题:(“移动”) or 主题:(“app”) or 主题:(“软件”) or 主题:(“程序”) or 主题:(“电脑”) or 主题:(“计算机”) or 主题:(“手机”) or 主题:(“远程”) or 主题:(“平台”)) AND (主题:(“干预”) or 主题:(“预防”) or 主题:(“治疗”) or 主题:(“疗法”) or 主题:(“医疗”) or 主题:(“管理”) or 主题:(“护理”) or 主题:(“支持”) or 主题:(“教育”) or 主题:(“咨询”) or 主题:(“缓解”) or 主题:(“监管”) or 主题:(“问诊”) or 主题:(“健康”))

**VIP Database:**

R=(产前 or 分娩 or 出生 or 怀孕 or 孕期 or 孕妇 or 孕产妇 or 妊娠 or 产妇 or 围产期 or 围生期 or 围生产期 or 产后 or 产褥期) AND R=(抑郁 or 抑郁症 or 忧郁 or 心理 or 情绪 or 心境) AND R=(网络 or 互联网 or 网页 or 网站 or 线上 or 在线 or 电子 or 移动 or app or 软件 or 程序 or 电脑 or 计算机 or 手机 or 远程 or 平台) AND R=(干预 or 预防 or 治疗 or 疗法 or 医疗 or 管理 or 护理 or 支持 or 教育 or 咨询 or 缓解or 监管 or 问诊 or 健康)
