# Supplementary material for: Translating Research Evidence Into Marketplace Application: Cohort Study of Internet-Based Intervention Platforms for Perinatal Depression
Source: J Med Internet Res. 2023 Apr 17;25:e42777. doi: 10.2196/42777 (PMC10152328; doi:10.2196/42777)
Supplement: Multimedia Appendix 2 [file jmir_v25i1e42777_app2.docx]

# Multimedia Appendix 2. *Quality assessment checklist.*

| **APA’ s dimension** | **Items for quality assessment** |
| --- | --- |
| **Background and Access** | Does the app identify ownership? |
|  | On which platforms/operating systems does it work? |
|  | Does the app work with accessibility features of the iPhone/android? |
|  | Has the app been updated in the last 6 months? |
|  | Are there additional or hidden costs? |
|  | Does is claim to be medical? |
| **Privacy and Security** | Is there a transparent privacy policy that is clear and accessible before use? (privacy policy; terms of use) |
|  | Does the app collect, use, and/or transmit sensitive data? |
|  | Does the app declare data use and purpose? |
|  | What third parties does the app share data with? |
|  | If appropriate, is the app equipped to respond to potential harms or safety concerns? |
| **Clinical Foundation** | Is there evidence of specific benefit from academic institutions, publications, end user feedback, or research studies? |
| **Usability** | Does the app clearly define functional scope? (Intervention methods; Human resources for supporting interventions; Whether to provide feedback to users; Whether to have a mood assessment) |
|  | What are the main engagement styles of the app? |
| **Data Integration towards Therapeutic Goal** | If intended to be used with a provider, does the app have the ability to export or transfer data? |
|  | Does the app improve therapeutic alliance between patient and provider? (Whether provided referral information; Whether to integrate user data into the healthcare system) |
