# Supplementary material for: Translating Research Evidence Into Marketplace Application: Cohort Study of Internet-Based Intervention Platforms for Perinatal Depression
Source: J Med Internet Res. 2023 Apr 17;25:e42777. doi: 10.2196/42777 (PMC10152328; doi:10.2196/42777)
Supplement: Multimedia Appendix 3 [file jmir_v25i1e42777_app3.docx]

# Multimedia Appendix 3. *Platform database.*

| **Authors** | **Year** | **Type of platforms** | **Name of platforms** |
| --- | --- | --- | --- |
| Ricardo F.Muñoz | 2007 | Website | Mamás y Bebés/Mothers and Babies |
| Alinne Z Barrera | 2015 |  |  |
| Lisa B Sheeber | 2012 | Website | Mom-Net |
| O’Mahen | 2013 | Website | Netmums |
| O’Mahen | 2014 |  |  |
| Brian G Danaher | 2013 | Website | MomMoodBooster |
| Cara L Solness | 2021 |  |  |
| Silje Marie Haga | 2013 | App | Mamma Mia |
| Filip Drozd | 2015 |  |  |
| Silje Marie Haga | 2019 |  |  |
| Silje Marie Haga | 2021 |  |  |
| Sona Dimidjian | 2014 | Website | Mindful Mood Balance |
| Jennifer N.Felder | 2017 |  |  |
| Anne H Salonen | 2014 | Website | Vauvan kaa |
| Salla Muuraiskangas | 2015 | App | Oiva |
| Nicole E. Pugh | 2016 | Website | Maternal Depression Online |
| Giulia Corno | 2016 | Website | Positive Pregnancy |
| Giulia Corno | 2018 |  |  |
| Jeannette Milgrom | 2016 | Website | MumMoodBooster |
| Yafit Hirshler | 2021 |  |  |
| Amit Baumel | 2016 | Website & App | 7 Cups |
| Amit Baumel | 2018 |  |  |
| Lori Wozney | 2017 | Website | Strongest Families™Managing Our Mood (MOM) |
| Erik Forsell | 2017 | Website | Internetpsykiatri |
| Liisa Hantsoo | 2017 | App | Ginger Emotional Support |
| Adriana Dyurich | 2017 | App | Veedamom |
| Ana Fonseca | 2018 | Website | Be a mom |
| Ana Fonseca | 2019 |  |  |
| Ana Fonseca | 2020 |  |  |
| Fabiana Monteiro | 2020 |  |  |
| Fabiana Monteiro | 2021 |  |  |
| Julia A Dalton | 2018 | App | Health-e Babies |
| Anne Moorhead | 2018 | App | Moment health |
| Raymond Bond | 2019 |  |  |
| Veena Prasad | 2018 | App | Veedamom |
| Adriana Dyurich | 2020 |  |  |
| Jennifer Duffecy | 2019 | Website | Sunnyside |
| Eva S Potharst | 2019 | Website | Mindful-ouderschap |
| Siobhan A Loughnan | 2019 | Website | MUMentum |
| Siobhan A Loughnan | 2019 |  |  |
| Ko Ling Chan | 2019 | App | iParent |
| Kevin Doherty | 2019 | App | Brightself |
| Alyssa Sawyer | 2019 | App | eMums Plus |
| Jennifer Huberty | 2020 | Website | Udaya |
| Hanna M Heller | 2020 | Website | MamaKits online |
| Maren Goetz | 2020 | App | Mindmom |
| Nazanin Jannati | 2020 | App | HAppy Mom |
| Lyndsay A Avalos | 2020 | Website & App | Headspace |
| Ai Kubo | 2021 |  |  |
| Claudia Carissoli | 2021 | App | BenEssere Mamma |
| Yaoyao Sun | 2021 | App | Spirit Healing |
| Katherine J Gold | 2021 | Website & App | Babycenter |
| Nicole Reilly | 2021 | Website & App | Mummatters |
