# Supplementary material for: Translating Research Evidence Into Marketplace Application: Cohort Study of Internet-Based Intervention Platforms for Perinatal Depression
Source: J Med Internet Res. 2023 Apr 17;25:e42777. doi: 10.2196/42777 (PMC10152328; doi:10.2196/42777)
Supplement: Multimedia Appendix 4 [file jmir_v25i1e42777_app4.doc]

**Multimedia Appendix 4.** *The associations between functional adjustments and survival time of internet-based PND intervention platforms (N=19).*

| **Functional adjustments** |  | **Survival time≤10 years (n=8), n (%)** | **Survival time＞10 years (n=11), n (%)** | ***P* value** |
| --- | --- | --- | --- | --- |
| **Intervention method** | No adjustment(s) | 5 (62.5) | 3 (27.2) | .44 |
|  | Reduce | 2 (25.0) | 4 (36.4) |  |
|  | Add | 1 (12.5) | 4 (36.4) |  |
| **Target population** | No adjustment(s) | 5 (62.5) | 4 (36.4) | .37 |
|  | Add | 3 (37.5) | 7 (63.6) |  |
| **Human support for interventions** | No adjustment(s) | 4 (50.0) | 6 (54.5) | .26 |
|  | Reduce | 1 (12.5) | 0 (0.0) |  |
|  | Add | 3 (37.5) | 2 (18.2) |  |
|  | Change | 0 (0.0) | 3 (27.2) |  |
| **Mood monitoring and assessment** | No adjustment(s) | 6 (75.0) | 5 (45.5) | .43 |
|  | Reduce | 1 (12.5) | 1 (9.0) |  |
|  | Add | 1 (12.5) | 5 (45.5) |  |
| **Synchronous communication** | No adjustment(s) | 7 (87.5) | 8 (72.7) | 1.00 |
|  | Reduce | 1 (12.5) | 2 (18.2) |  |
|  | Add | 0 (0.0) | 1 (9.1) |  |
| **Platform type** | No adjustment(s) | 8 (100.0) | 9 (81.8) | 1.00 |
|  | Add | 0 (0.0) | 1 (9.1) |  |
|  | Change | 0 (0.0) | 1 (9.1) |  |
