# Supplementary material for: Translating Research Evidence Into Marketplace Application: Cohort Study of Internet-Based Intervention Platforms for Perinatal Depression
Source: J Med Internet Res. 2023 Apr 17;25:e42777. doi: 10.2196/42777 (PMC10152328; doi:10.2196/42777)
Supplement: Multimedia Appendix 5 [file jmir_v25i1e42777_app5.docx]

# Multimedia Appendix 5. *Quality assessment of internet-based PND intervention platforms against APA’ s model (N =19).*

| **APA’ s dimension** | **Items** |  | **Platform (n=19)** |
| --- | --- | --- | --- |
| **Background and Access** | Platform developer | Private company | 5 |
|  |  | Academic organization | 5 |
|  |  | Medical health provider | 3 |
|  |  | Multiple type of developers | 6 |
|  | Platform type | Website only | 11 |
|  |  | App only | 5 |
|  |  | Both Website and App | 3 |
|  | Application compatibility^a^ | iOS devices | 1 |
|  |  | Android devices | 1 |
|  |  | iOS & Android devices | 6 |
|  | Whether updated in the last 6 months | Yes | 6 |
|  |  | No | 4 |
|  |  | Unknown | 9 |
|  | Whether declared to charge | Yes | 7 |
|  |  | No | 12 |
|  | Whether claimed to be medical | Yes | 1 |
|  |  | No | 18 |
| **Privacy and Security** | Whether had privacy policy | Yes | 14 |
|  |  | No | 5 |
|  | Whether had terms of use | Yes | 13 |
|  |  | No | 6 |
|  | Whether claimed to collect, use and/or transmit users’ data | Yes | 16 |
|  |  | No | 3 |
|  | Whether declared data use and purpose | Yes | 16 |
|  |  | No | 3 |
|  | Whether used external resources from third parties | Yes | 13 |
|  |  | No | 6 |
|  | Whether had crisis management mechanisms | Yes | 8 |
|  |  | No | 11 |
| **Clinical Foundation** | Whether cited relevant research-based information | Yes | 8 |
|  |  | No | 11 |
| **Usability** | Intervention method | Health education | 17 |
|  |  | Mindfulness/meditation | 6 |
|  |  | Cognitive behavioral therapy (CBT) | 5 |
|  |  | Social support (family, peer) | 5 |
|  |  | Diary | 3 |
|  | Human support for interventions | Professionals | 6 |
|  |  | Non-professionals | 5 |
|  |  | Mixture of both types of human support | 2 |
|  |  | None | 6 |
|  | Whether to provide feedback to users | Yes | 10 |
|  |  | No | 9 |
|  | Whether to have a mood assessment | Yes | 10 |
|  |  | No | 9 |
|  | Platform engagement styles | image-text only | 4 |
|  |  | image-text plus (with audio/video) | 14 |
|  |  | image-text plus + AI | 1 |
| **Data Integration towards Therapeutic Goal** | Whether data can be exported | Yes | 10 |
|  |  | No | 9 |
|  | Whether provided referral information | Yes | 6 |
|  |  | No | 13 |
|  | Whether to integrate users' data into the healthcare system | Yes | 2 |
|  |  | No | 17 |

a. only suitable for platforms with App (n=8)
