# Supplementary material for: Translating Research Evidence Into Marketplace Application: Cohort Study of Internet-Based Intervention Platforms for Perinatal Depression
Source: J Med Internet Res. 2023 Apr 17;25:e42777. doi: 10.2196/42777 (PMC10152328; doi:10.2196/42777)
Supplement: Multimedia Appendix 6 [file jmir_v25i1e42777_app6.doc]

**Multimedia Appendix 6.** *The associations between quality indices and survival time of internet-based PND intervention platforms (N=19).*

| **APA’ s dimension** | **Items** |  | **Survival time≤10 years** **(n=8), n (%)** | **Survival time＞10 years (n=11), n (%)** | ***P* value** |
| --- | --- | --- | --- | --- | --- |
| **Background and Access** | Platform developer | Private company | 2 (25.0) | 3 (27.3) | 1.00 |
|  |  | Academic organization | 2 (25.0) | 3 (27.3) |  |
|  |  | Medical health provider | 1 (12.5) | 2 (18.1) |  |
|  |  | Multiple type of developers | 3 (37.5) | 3 (27.3) |  |
|  | Platform type | Website only | 4 (50.0) | 7 (63.6) | .15 |
|  |  | App only | 4 (50.0) | 1 (9.1) |  |
|  |  | Both Website and App | 0 (0.0) | 3 (27.3) |  |
|  | Whether updated in the last 6 months | Yes | 1 (12.5) | 5 (45.5) | .25 |
|  |  | No | 3 (37.5) | 1 (9.0) |  |
|  |  | Unknown | 4 (50.0) | 5 (45.5) |  |
|  | Whether declared to charge | Yes | 3 (37.5) | 4 (36.4) | 1.00 |
|  |  | No | 5 (62.5) | 7 (63.6) |  |
|  | Whether claimed to be medical | Yes | 0 (0.0) | 1 (9.1) | 1.00 |
|  |  | No | 8 (100.0) | 10 (90.9) |  |
| **Privacy and Security** | Whether had privacy policy | Yes | 5 (62.5) | 9 (81.8) | .60 |
|  |  | No | 3 (37.5) | 2 (18.2) |  |
|  | Whether had terms of use | Yes | 6 (75.0) | 7 (63.6) | 1.00 |
|  |  | No | 2 (25.0) | 4 (36.4) |  |
|  | Whether claimed to collect, use and/or transmit users’ data | Yes | 6 (75.0) | 10 (90.9) | .55 |
|  |  | No | 2 (25.0) | 1 (9.1) |  |
|  | Whether declared data use and purpose | Yes | 6 (75.0) | 10 (90.9) | .55 |
|  |  | No | 2 (25.0) | 1 (9.1) |  |
|  | Whether used external resources from third parties | Yes | 3 (37.5) | 10 (90.9) | .04 |
|  |  | No | 5 (62.5) | 1 (9.1) |  |
|  | Whether had crisis management mechanisms | Yes | 2 (25.0) | 6 (54.5) | .35 |
|  |  | No | 6 (75.0) | 5 (45.5) |  |
| **Clinical Foundation** | Whether cited relevant research-based information | Yes | 1 (12.5) | 7 (63.6) | .06 |
|  |  | No | 7 (87.5) | 4 (36.4) |  |
| **Usability** | Intervention method | Psychotherapy (CBT, BA, PST etc.) | 1 (12.5) | 4 (36.4) | .34 |
|  |  | Non-psychotherapy | 7 (87.5) | 7 (63.6) |  |
|  | Human support for interventions | Yes | 4 (50.0) | 9 (81.8) | .32 |
|  |  | No | 4 (50.0) | 2 (18.2) |  |
|  | Whether to provide feedback to users | Yes | 3 (37.5) | 7 (63.6) | .37 |
|  |  | No | 5 (62.5) | 4 (36.4) |  |
|  | Whether to have a mood assessment | Yes | 3 (37.5) | 7 (63.6) | .37 |
|  |  | No | 5 (62.5) | 4 (36.4) |  |
|  | Platform engagement styles | image-text only | 0 (0.0) | 4 (36.4) | .06 |
|  |  | image-text plus (with audio/video) | 8 (100.0) | 6 (54.5) |  |
|  |  | image-text plus + AI | 0 (0.0) | 1 (9.1) |  |
| **Data Integration towards Therapeutic Goal** | Whether data can be exported | Yes | 3 (37.5) | 7 (63.6) | .37 |
|  |  | No | 5 (62.5) | 4 (36.4) |  |
|  | Whether provided referral information | Yes | 3 (37.5) | 3 (27.3) | 1.00 |
|  |  | No | 5 (62.5) | 8 (72.7) |  |
|  | Whether to integrate users' data into the healthcare system | Yes | 1 (12.5) | 1 (9.1) | 1.00 |
|  |  | No | 7 (87.5) | 10 (90.9) |  |
